# Predictive Factors of Treatment Resistance in First Episode of Psychosis: A Systematic Review

**DOI:** 10.3389/fpsyt.2019.00067

**Published:** 2019-02-26

**Authors:** Paola Bozzatello, Silvio Bellino, Paola Rocca

**Affiliations:** Department of Neuroscience, University of Turin, Turin, Italy

**Keywords:** schizophrenia, first episode of psychosis, treatment resistance, non-response, predictors of response, clinical factors, biological factors

## Abstract

**Background:** Clinical and functional outcome improvement in psychotic disorders is a challenge for the investigators. Recent advances offered opportunities for ameliorating the course of the illness during its early stages and for identifying treatment-resistant patients. Patients who had not response to two different antipsychotics, administered at correct doses for a sufficient period, can be operationally considered treatment-resistant. Available evidence suggested that the response's trajectory to the antipsychotic treatment revealed that a small proportion of subjects are poor responders (8.2%), the majority of patients have a moderate response (76.4%), and only 15.4% can be considered rapid responders with the greatest magnitude of response. Patients with first episode of psychosis generally obtain a more favorable response profile. Nevertheless, in around 25% of these patients symptoms of psychosis persist with a worse long-term course of illness.

**Objectives:** The aim of this review is to report current evidences on the main predictors of treatment non-response in patients at early stage of psychosis.

**Methods:** We used a specific string that guaranteed a high sensitive search in pubmed. We included the following types of publications: randomized-controlled trials, observational studies, longitudinal studies, retrospective studies, case-control studies, open-label investigations, cohort studies, and reviews. Publications must concern predictors of treatment resistance in early psychosis.

**Results:** Forty-seven records were included: 5 reviews, 3 meta-analyses, 22 longitudinal studies, 2 retrospective studies, 1 naturalistic study, 6 randomized controlled trials, 2 open-label studies, 2 case-control studies, 4 cohort studies, 2 retrospective studies. Several factors were identified as predictors of treatment resistance: lower premorbid functioning; lower level of education; negative symptoms from first psychotic episode; comorbid substance use; younger age at onset; lack of early response; non-adherence to treatment; and longer duration of untreated psychosis. The role of gender and marital status is still controversial. More evidences are needed about neurobiological, genetic, and neuroimaging factors.

**Conclusions:** The identification of specific predictive factors of treatment resistance in patients with first episode of psychosis ameliorates the quality of clinical management of these patients in the critical early phase of schizophrenia.

## Introduction

The point prevalence estimated for schizophrenia is around 0.6–0.8% and the lifetime prevalence is about 1%. In general, first psychotic episode starts in young adulthood, but the onset of the disorder is preceded by a variety of prodromal symptoms (1–4). There is a general accordance among investigators that treatment response in schizophrenia is very heterogeneous (5, 6). “Treatment outcome has been extensively studied in first-episode schizophrenia. However, the majority of investigations have mainly focused on favorable outcome measures such as response, remission and recovery” (7). Although a good number of patients obtain the remission of symptoms, a significant percentage of cases remains “actively and persistently psychotic despite correct pharmacological treatments” (8). Nevertheless, whether the resistance to treatments is present from the onset of illness (first episode of psychosis-FEP) or whether patients gradually become resistant due to the disease progress is still little known today (8). Some authors state that “patients with FEP may show long-term incomplete remission or treatment resistance in a percentage ranged between 10 to 50%” (9–11). Outcomes in first-episode psychosis (FEP) vary on a continuum from complete remission and full recovery to complete failure of response or treatment resistance. A possible reason of this variability is the intrinsic diagnostic instability of patients at first episode of psychosis.

Resistance to treatments represent a critical topic in schizophrenia spectrum disorders as it is linked with an higher risk of a clinical deterioration, hospitalizations, chronicity, neurotoxic effects of relapses, suicide, aggressive conducts, poor quality of life, and low level of real-world functioning (7, 8, 12–16). Clinical, social, and vocational recovery failure increases the economic cost and enhances burden for family members and stigma for patients (11). Available evidences suggest that the trajectory of response to the antipsychotics treatment reveals that a small proportion of subjects are poor responders (8.2%), the majority of patients have a moderate response (76.4%), and only 15.4% can be considered rapid responders with the greatest magnitude of response (17). Patients with FEP generally obtain a more favorable response profile than patients after multiple episodes. Nevertheless, in around 25% of these patients symptoms of psychosis persist with a worse long-term course of illness (17–22). The precocious identification of individuals who fail to respond to initial interventions may ameliorate the treatment approach at an earlier phase of illness to avoid multiple, unnecessary switches or repeated medication trials and to prevent accruing morbidity. Specialized integrated early interventions, including antispychotics, individual psychological treatment, family, and vocational support are shown to be effective to improve treatment response (23). Unfortunately, as regards the predictive factors of treatment resistance in early phase of illness, the literature to date is still sparse and inconclusive. The present review is aimed to provide an updated overview of current evidences on the main predictive factors of non-response and treatment resistance in patients at early stage of psychosis.

## Treatment non-response and Treatment-Resistance: Definitions

Investigations indicated that response to antipsychotic treatments begins in the first weeks of treatment with the largest effect in reducing symptoms in the first 2 weeks (17, 24). Remission was defined as “a state, of at least 6 months' duration, in which no symptoms or only mild symptoms, not interfering with daily functioning, were experienced” (25). Early non-response was operationally defined as “ <20% improvement on Positive and Negative Symptoms Scale (PANSS) or Brief Psychiatric Rating Scale (BPRS) total score at 2 weeks” (26). Some authors suggested that “patients who have not a minimal improvement after 2 weeks of treatment are unlikely to respond at a later phase and may benefit from a drug change” (26, 27).

Kane et al. (28) defined treatment resistance with three criteria. “First, the patient fails to respond to three or more adequate trials of antipsychotic treatment within the last 5 years, including antipsychotics of two distinct classes at dose greater than or equal to the equivalent of 1,000 mg/day of chlorpromazine.” Moreover, it is widely accepted that three or more second generation antipsychotics failures define treatment resistance (29). “Second, at least two of the symptoms of conceptual disorganization, suspiciousness, hallucinations, and unusual thought content persist with a score at least moderate in severity. Lastly, patient has evidence of substantial symptoms despite current optimized treatment to which the patient is adherent, defined as a score ≥45 on the BPRS or ≥90 on the PANSS” (28).

In line with the National Institute for Health and Care Excellence (NICE) (2) criteria, “patients who had received two sequential antipsychotic trials, each of at least 4 weeks at a daily dose of 400–600 mg of chlorpromazine equivalents, but continued to have persistent psychotic symptoms, which was defined as having a rating of at least moderate severity on one or more positive symptoms, and despite recorded adherence to medication, were classified as treatment resistant. Patients were classified as treatment resistant at onset if they met criteria for treatment resistance following the first two trials with antipsychotics” (2).

Although the definition of the treatment resistance is mainly centered on clinical symptoms' relief, several authors suggested to include the evaluation of psychosocial elements, such as adherence to medications, and daily functional outcome in the context of resistance definition. In this view, patients can be considered resistant to therapy only if both clinical and functional outcome are compromised (30–32).

A recent review performed by the treatment-resistant schizophrenia working group consensus guidelines (33) concluded that there was a relative consensus among authors in defining subjects treatment-resistant: a confirmed diagnosis of schizophrenia based on validated criteria, an adequate pharmacological treatment, and the persistence of significant symptoms despite adequate treatment (33). Although significant differences exist among the main guidelines for the treatment of schizophrenia in terms of operationalized definition of resistance, some commonalities can be observed. In particular, the shared criteria concern the requirements for at least two failed treatment trials, each of a minimum of 6 weeks, and the use of standardized rating scales (2, 34–38). In addition, the working group consensus guidelines (33) suggested to incorporate into criteria for defining treatment-resistance two further elements: patients adherence and functional impairment.

## Methods

In April 2018, we performed an electronic search in PubMed on predictive factors of treatment resistance in FEP, with no filter or MESH restriction, using the following search string: “schizophrenia” OR “psychosis” OR “first-episode of psychosis” OR “early psychosis” AND “predictive factors” OR “predictors of response” AND “resistant patients” OR “treatment resistance” OR “treatment non-response.” This string provided a high specific search, obtaining an accurate selection of article indexed in PubMed. We included the following types of publications: randomized-controlled trials, observational studies, longitudinal studies, retrospective studies, case-control studies, open-label investigations, cohort studies, and reviews until July 2018. Publications must concern predictors of treatment resistance in early psychosis as the principal issue (all definitions of resistance described in the previous paragraph are included in our revision). We excluded publications written in languages different from English.

## Results

The search described in the previous section provided 1208 records. Eighty additional records were identified from another research platform (Google scholar). We removed the duplicates records (246). Eligibility status for all retrieved articles was determined in two stages. First, all studies were screened basing upon title and abstract. Second, papers passing the initial title and abstract screen were reviewed basing upon the full manuscript. Nine hundred seventy-seven records were excluded because they did not fit the objective of the review, 11 as the full text was not available. Full text articles selected for eligibility were 54; seven of them were excluded as were not written in English. This review included 47 records: 5 reviews, 3 meta-analyses, 22 longitudinal studies, 2 retrospective studies, 1 naturalistic study, 6 randomized controlled trials, 2 open-label studies, 2 case-control studies, 4 cohort studies.

**Figure 1:**
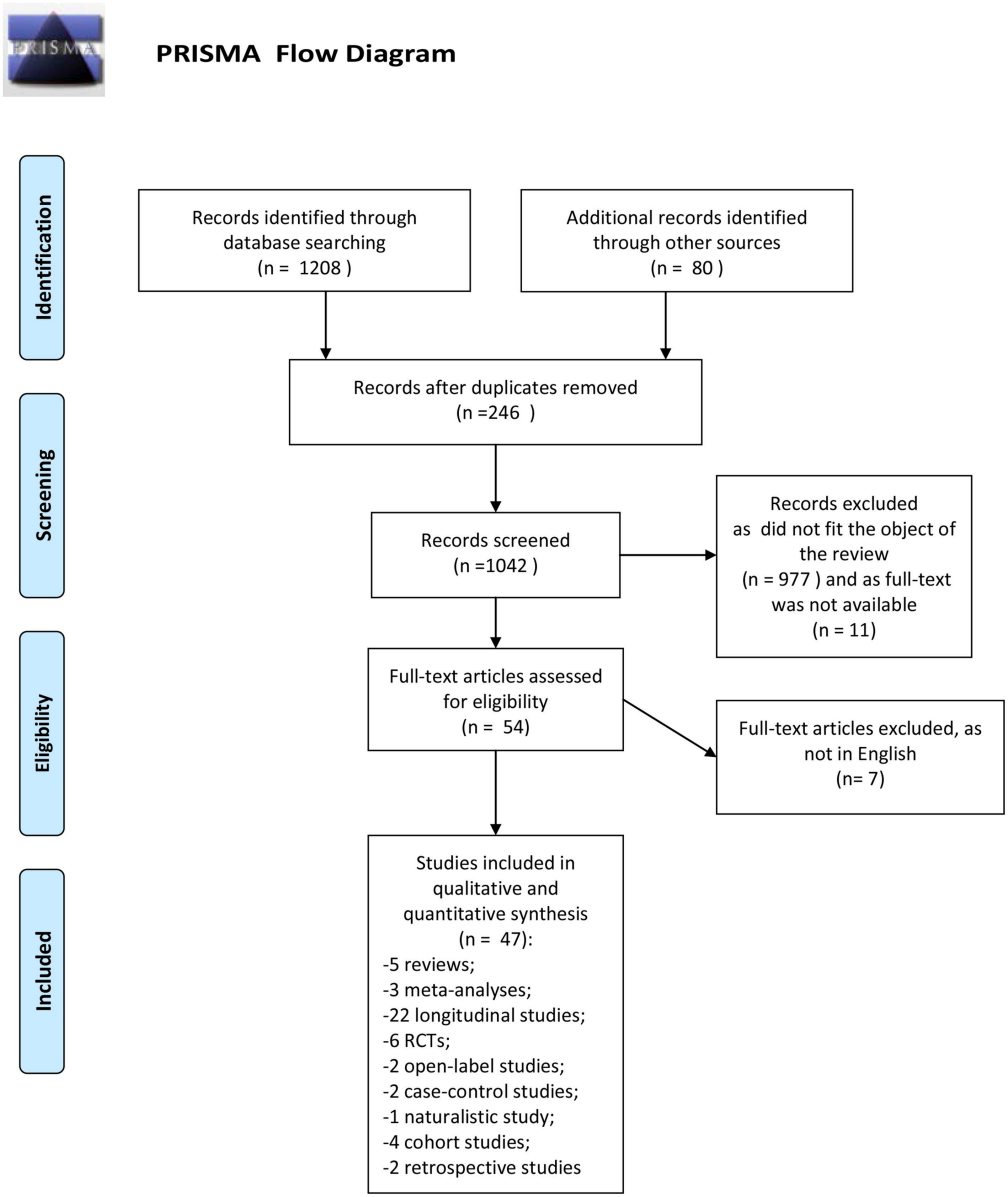
PRISMA flow diagram. Adapted from Moher et al. (39).

Number of studies participants was ranged between 56 and 1,175. All studies included both genders; the majority of studies had an equal distribution of males and females. The predominant ethnicity was the Caucasian. Duration of the longitudinal studies was ranged between 1 and 10 years, while the duration of controlled-trials was ranged between 6 weeks (acute phase) and 1 year. Ninety percent of studies enrolled participants at early stage of illness (first episode of psychosis, first hospital contact, recent onset of psychosis). All subjects presented a schizophrenia spectrum disorder. The PRISMA flow chart of this review is presented in Figure 1.

### Patient-Related Predictors of Treatment Resistance

Predictors of treatment resistance that are linked to patients characteristics were investigated in recent years. In particular age, gender, premorbid functioning, level of education, and marital status are the main individual-related factors that were studied in the context of resistance to treatment.

Crespo-Facorro et al. (40) performed a randomized controlled study, in which 172 patients with a first episode of non-affective psychosis were assigned to haloperidol, olanzapine, and risperidone in a random way. Results concerned the “6-weeks acute phase of a large epidemiological and longitudinal (3 years) intervention program of first-episode psychosis” (40). Among the patient-related variables, authors found that lower premorbid functioning was one of the most important factors in distinguishing antipsychotic non-responders from responders. This result was confirmed in a second study conducted by the same authors (41) on 375 FEP subjects. Similar findings were also reported by Addington and Addington (42) in 240 schizophrenia spectrum disorder patients with FEP in a period of follow-up of 36 months, and by Albert et al. (43) in a 5-years study including 255 FEP patients. Several investigations adequate for sample characteristics and duration (44–47) suggested that a good premorbid adjustment and social environment may predict a better response to treatments. Recently, Lasalvia et al. (48) confirmed this result stating that “premorbid adjustment and insight predicted outcome regardless of the kind of treatment” (48).

Wimberley et al. (49) performed a 9-years cohort study (population-based) in 8624 patients with a diagnosis of schizophrenia to identify predictive factors of treatment resistance at first hospital contact. Results showed that “a younger age, living in a less urban area, and primary education level were all significantly associated with treatment-resistant schizophrenia” (49). The relationship between lower educational level and treatment resistance was previously found by other three studies performed by Verma et al. (50), Díaz et al. (51), and Lasalvia et al. (48) in samples of respectively, 1,175 subjects with schizophrenia spectrum disorders, 174 patients with FEP, and 444 patients with schizophrenia. In a recent study published by Di Capite et al. (52) evaluating predictors of relapse in 63 patients with first-episode psychosis who have discontinued antipsychotic medications in a period on 1 year, authors concluded that “to be engaged in education or training was not predictive of relapse” (52).

Only few studies found a relationship between age and treatment-response, also due to the fact that many investigations have not considered age but age at illness onset. Nevertheless, two studies (43, 53) reported an association between older age and remission. Study published by Zhang et al. lasted 1 year and included 398 patients never medicated with FEP in schizophrenia spectrum disorders. The role of gender, in particular male gender, as predictor of worse response to treatments since the FEP is still controversial, although male gender is traditionally retained an indicator of poor outcome in schizophrenia. Some studies actually suggested that male gender may be considered a predictor of treatment non-response in FEP and schizophrenia spectrum disorders. In particular, Selten et al. (54) performed a study with a follow-up phase lasting 30 months in 125 subjects with FEP and schizophrenia spectrum disorders diagnosis. They found that the predominant predictor of poor outcome was male gender (together with substance abuse). Similar findings were reported by Derks et al. (55) and Díaz et al. (51). The two study had a similar design, the same duration of 1 year and the same criteria of inclusion: patients with FEP in schizophrenia spectrum disorders, including brief reactive psychosis and schizoaffective disorder. The only significant difference concerned the sample size, as in the first study were included 498 patients, while in the second they were only 174. Lower odds for remission were found in male patients in both studies. These findings were confirmed by the study performed by Di Capite et al. (52) that suggested that males had a higher risk of relapse after antipsychotic discontinuation then females and in the study conducted by Lally et al. (56) in 246 FEP patients with schizophrenia spectrum disorders concluding that treatment resistance was strictly connected with male sex. Other investigations considered the female gender as one of predictive factors of response to treatment. There was a broad accordance among authors in retaining that the female sex represents a strong predictor of remission and recovery (43, 50, 57). However, it must be noticed that many other studies have not confirmed the effect of gender on response to treatments (15, 19, 21, 44, 47, 58–64).

Data concerning the role of marital status in predicting treatment resistance are scarce and heterogeneous. Emsley et al. (45) found in a study of 1 year on 57 patients with FEP and schizophrenia spectrum disorders a significant relationship between single status and resistance to treatments. In a similar way Díaz et al. (51) reported that single status predicted non-response and non-remission. On the contrary, Teferra et al. (65) reported that single status may predict better outcome in a 5-years study performed in Ethiopia in 312 patients with schizophrenia.

### Disorder-Related Predictors of Treatment Resistance

With respect to clinical predictors of treatment resistance in FEP several symptomatic factors have been discussed about treatment resistance and poor long-term outcome. Positive symptoms were thought for long time to be the most important outcome measure and were the standard parameters for treatment resistance assessment. This was due to the fact that other symptoms were not correctly recognized or undervalued, or symptoms such as negative symptoms were considered unresponsive to treatment.

Considering the clinical response only in terms of the positive symptoms decrease is clearly reductive. In fact, schizophrenia since its early stages includes a wider spectrum of symptoms, involving negative, cognitive, and/or disorganized symptoms, as well as functional deficits. Several authors have shown that residual positive symptoms and global psychopathology, cognitive impairment, and enduring negative symptoms constituted the indicators of the severity of schizophrenia and were associated with non-response to antipsychotics (66–68).

In an epidemiological cohort study lasting 18 months and including 367 FEP patients “treated with olanzapine or risperidone” (69), authors found that 33% of patients with schizophrenia had continuous positive symptoms and another 22% presented positive symptoms following relapse. Overall, 35% of patients were found to be in symptomatic remission at 18 months but 20% had persistent psychoses with an unchanged severity of illness. Crespo-Facorro et al. (41) found that the presence of positive and disorganized symptoms at baseline predicted resistance to treatment. Addington and Addington (42) stated that “the high level of both positive and negative symptoms may predict poor outcome in schizophrenia spectrum disorders.”

Investigations focused on the evaluation of positive symptoms of schizophrenia as poor response and resistance predictors remain rather sparse. However, on this topic, an interesting 10-year follow-up study has been performed to investigate “long-term trajectories of positive and negative symptoms in FEP” (70). Four-hundred-ninety-six patients with diagnosis of schizophrenia spectrum disorders were assessed with several evaluation tools, such as the Scales for the Assessment of Positive (SAPS) and Negative Symptoms (SANS) (71). Results indicated that around 60% of subjects experienced a reduction followed by a stabilization of positive symptoms during a period ranged between one and 5 years, while changes in negative symptoms did not reach the same degree. Moreover, in patients who responded to treatment the trend of positive symptoms continued to improve across 10 years. On the other hand, 50% of the cohort did not obtain a reduction of negative symptoms over the 10 years (70). Individuals who persistently suffer from negative symptoms may present impaired functioning and psychological outcomes with a higher rates of treatment resistance in comparison with people who show a decrease of negative symptoms over time (72, 73). As negative symptoms are already present and prominent at the early phase of the illness, in a minority of patients the full syndrome of treatment resistance is present since the FEP. Milev et al. (67) performed a longitudinal first-episode study with a 7-year follow-up on 99 subjects who were in their first episode of illness. Authors found a significant influence of both cognitive and negative symptoms on response to treatments. Similar findings were observed by Siegel et al. (74) in 208 patients with schizophrenia monitored for 3 years.

Higher severity of negative symptoms at the beginning of the trials was recognized to be a powerful predictor of resistance to treatments also in more recent investigations. In particular, Ventura et al. (75) showed that the degree of negative symptoms in 149 recent-onset (1 year) subjects with schizophrenia was associated with impaired everyday functioning 7 years later. Negative symptoms in early psychosis did not change in the first year and predicted social functioning after 12 months. In addition, negative symptoms at onset of schizophrenia were related to the persistence of negative symptoms after 8 years. These results suggested that “negative symptoms may be an important early course target for interventions to promote the recovery” (75). Demjaha et al. (8) performed a longitudinal study in a large cohort of 323 FEP patients that were studied for 10 years of follow-up. Findings showed that the strongest effect on treatment resistance was exercised by the negative symptoms at onset of illness. Other predictors of non-response and resistance in this study were the younger age of onset and the diagnosis of schizophrenia. Yoshimura et al. (76) confirmed the previous results about the influence of negative symptoms on resistance in 131 patients with schizophrenia. Investigation conducted by Downs et al. (77) in 638 subjects with early-onset psychosis highlighted the importance of the negative symptomatology in predicting response also in particular populations such as children and adolescents. In fact, authors concluded that early psychosis is characterized by negative symptoms that significantly contributed to the unsuccessful response or resistance to treatment. Other investigators evaluated the predictors of remission in schizophrenia spectrum disorders. Some of them (78) observed that a lower degree of positive, negative, and general symptoms was linked with remission, while other (62) found that only a lower degree of negative symptoms at baseline was responsible of a better response to treatment. Cognitive performances and disorganized symptoms obtained less attention among investigators and few studies have been performed on this issue. Chiliza et al. (7) concluded that both cognitive and disorganized symptomatology predicted resistance in 126 patients with schizophrenia spectrum disorders. Other Levine and Rabinowitz (47) identified only cognitive impairment at baseline as predictor of non-response to treatment in 49 FEP patients with schizophrenia spectrum disorders. This finding is in accordance a more recent study (16) in which authors compared resistant and responder schizophrenic patients and observed that resistant subjects had more severe cognitive impairment than responders, in particular in verbal memory tasks.

Two illness-related factors that received particular attention in the context of resistance to treatment are the diagnosis of schizophrenia and the age at onset of disease. Some studies considered both these factors as predictors of non-response (8, 41, 47). Other investigations found a significant association only between diagnosis of schizophrenia and treatment resistance (49, 70, 79).

Comorbidity is another clinical factor that we need to consider in this context. In particular, substance use disorders had a significant impact in terms of clinical manifestations and treatment outcome. Around 40% of individuals with schizophrenia spectrum disorders meet criteria for alcohol use, and about 30% for substance use disorders (80). To our knowledge only 5 studies evaluated how substance use may predict outcome in schizophrenia spectrum disorders. The first was a 30 months follow-up study (54) and involved 125 FEP patients with schizophrenia spectrum disorders. Results showed that the conjunction of male gender and substance abuse (cannabis) was a predominant predictor of non-response to treatment in this population. Pelayo-Terán et al. (81) confirmed these findings in a 6-weeks study on 161 FEP patients with schizophreniform and schizoaffective disorders, specifying that misuse of cannabis predicted non-response of both positive and negative symptoms. Studies performed by Austin et al. (70) and Wimberley et al. (49) confirmed the role of substance use disorders in treatment resistance in patients with FEP. The design of these investigations was previously described in this review.

Boter et al. (82) in a 12-months follow-up study considered this topic from another point of view and investigated predictors of remission in 498 FEP patients with schizophreniform or schizoaffective disorder. They identified the absence of substance use disorder as predictor of remission.

### Neurobiological Predictors of Treatment Resistance

One of the controversies in literature concerned whether psychosis onset derives by some neurobiological abnormalities or whether it exerts a long-term toxic effect on the brain *per se*. We have limited knowledge to identify which neurobiological factors allow to separate from the FEP responders and resistant patients. Anyway, some neurobiological and neuroimaging factors may be detected as potentially involved in the mechanisms of response/non-response to therapies. Some experts have suggested that patients with FEP present a variability in response to antipsychotics that is induced by different neurobiological correlates. One of the hypotheses concerns the relationship between the activity of dopamine system and treatment response. Some findings support this hypothesis as they found high levels of synthesis and release of dopamine in schizophrenic patients with a good response, in comparison with resistant subjects (83).

Some studies examined in plasma the level of dopamine metabolites and observed that a lower concentration before treatment is related to a less favorable response to first-line medications (84, 85). Moreover, a study conducted post-mortem compared two groups with positive and negative response to treatment and identified a lower number of dopaminergic synapses in patients with poor response.

Kim et al. (86) performed a small study including 12 patients with schizophrenia who received clozapine and were considered resistant, 12 patients who had considered responders, and 12 controls with no psychiatric diagnosis. Authors found that the subgroup of resistant patients were distinguished by reduced level of dopamine synthesis in striatum. This findings may suggest that some neurobiological factors may be responsible for treatment resistance in schizophrenia and a candidate biomarker of response is the level of that dopamine synthesis.

In another recent study (87) authors reported that a greater decrease of myelination in substantia nigra was observed in cases of schizophrenia with a poor response to treatment in comparison with responders and healthy controls. This finding does not allow to conclude that substantia nigra aberrations may be considered as predictors of treatment resistance. In fact, these aberrations could be explained at least in part as a consequence of toxicity of relapses and non-response to therapies. Nevertheless, this investigation indicates the effort of investigators to better understand, also in terms of neurobiological abnormalities the treatment resistance phenomena.

Alterations in the levels of cortisol and other markers of inflammations have been registered at the onset of psychosis. Mondelli et al. (88) performed a 12 weeks follow-up study on 68 FEP patients and 57 controls. Authors collected saliva and blood samples to measure the level of cortisol and serum markers of inflammations before and after antipsychotic administration. Results showed that blunted cortisol awakening response and increased proinflammatory cytokines were predictors of resistance in the early phases of psychosis. These factors are potentially considered as strong predictive factors of non-response in this phase of the illness.

Regulatory system of cortisol was already found implicated in schizophrenia treatment resistance in previous studies. For example, pituitary volume measured at the onset of psychotic disorders was investigated as a factor that can predict response in FEP (89). Authors evaluated if baseline pituitary volume was significantly related to treatment response in 42 FEP patients treated with quetiapine for 12 weeks. Results indicated that pituitary volume had an inverse relation with decrease of symptom severity. This association highlighted the relevance of hypothalamic-pituitary-adrenal axis in the early stages of psychosis.

In recent years, neuroimaging techniques have allowed to study if there is a relationship in psychotic disorders between refractoriness to therapies and structures of brain. Some investigators sustained that poor treatment response is significantly related to the diminished volume of gray matter (90). In one-hundred-twenty-six patients, including 80 subjects with a diagnosis of first-episode psychosis, gyrification was measured in multiple areas of brain and a significant hypogyria was found in comparison with 46 healthy subjects. In particular, subjects who did not respond to treatment showed hypogyria in bilateral insular regions, left frontal area, and right temporal area when compared with patients who responded. So, authors concluded that at first stages of illness non-responders had significant alterations of cortical folding compared with responders and with healthy subjects. Due to the scarcity of investigations on this field it is not possible to draw any conclusion. Nevertheless, it seems that the pituitary volume measured with structural magnetic resonance may represent a potential predictor of response/non-response in psychosis at onset.

### Treatment-Related Predictors of Resistance

In our opinion three factors related to treatment are particularly relevant in predicting resistance in schizophrenia and require to be mentioned and discussed: adherence, early response, and duration of untreated psychosis. Concerning adherence to therapies, antipsychotic treatment non-adherence was found tightly linked to low odds for response and remission, in particular in the first stages of illness (20, 22, 52, 91). Some Pelayo-Terán et al. (81) suggested that adherence was one of the most robust predictors of the first relapse. The main studies on this topic were in accordance to conclude that an higher level of adherence since FEP predicted response and remission of the illness (44, 53, 62, 82). In light of these considerations, prescription of long-acting antipsychotics in patients with several risk factors for relapse (for example diagnosis of schizophrenia, comorbid substances abuse, prominent negative symptoms, and non-adherence to oral antispychotics) also at first-episode of psychosis may significantly improve outcome of FEP (22). Response to antipsychotic treatments should begin within first weeks of therapy, with the greatest effect in the first 2 weeks (24, 92). Correll et al. (93) performed an open-label 4-weeks study in 131 patients with schizophrenia who received fluphenazine. They showed that poor response in the first week of treatment with a typical antipsychotic may predict non-response also at fourth week of the trial. The same results were obtained by Leucht et al. (94, 95) who analyzed data from 1708 patients with schizophrenia or schizophreniform disorder enrolled in 7 randomized controlled trials (RCT) on antipsychotics. In addition, these authors stated that minimal symptoms reduction at week 2 had high specificity and sensitivity in identifying responders at week 4. This datum was also confirmed by Samara et al. (27) in a meta-analysis of 34 studies aimed to evaluate the association between lack of symptoms improvement at week 2 and later non-response. Kinon and collaborators conducted two investigations (26, 96). In the first one, authors analyzed data from 5 double-blind RCTs including 1,077 patients with schizophrenia spectrum disorders who received second-generation antipsychotics. Authors considered a period of observation of 6 months and assessed at different time points medication discontinuation rates. Results showed that early non-response predicted subsequent lack of response, but early response could not be considered as a predictor of following response. The same Kinon et al. (26) performed a 12-weeks RCT aimed to investigate in 628 patients diagnosed with schizophrenia or schizoaffective disorder who initially received risperidone whether the early response (within 2 weeks) to an antipsychotic medication may predict the following response. Subjects who responded proceeded with risperidone, while patients who did not respond in the first 2 weeks were randomized to continue with risperidone or to receive olanzapine for other 10 weeks. Findings reported that early non-responders may require more than 4–6 weeks to respond to antipsychotics.

Another important variable associated with non-response and resistance is the duration of untreated psychosis (DUP). DUP is “the period between the time psychosis begins to the time adequate treatment is sought and secured. The mean duration of untreated psychosis is ranged between 1 and 2 years and the median is about 6 months” (97). A more prolonged DUP has been related to a longer time of response to treatment in patients who presented a first-episode of psychosis and to an impaired course of the disorder (21, 98–100). Bottlender et al. (101) conducted a long-term study lasting 15 years in 58 patients with schizophrenia followed-up since their first psychiatric admission. Authors observed that a higher level of negative, positive and general psychopathological symptoms and a lower global functioning 15 years after the first psychiatric admission were associated with a prolonged DUP. In accordance with these results, another 10-years follow-up study (70) concluded that a longer DUP predicted worse trajectories of positive and negative symptoms in time, with a poor response to medications. Friis et al. (79) stated that first-episode psychosis patients that have not begun an adequate antipsychotic treatment at least within 6 months (having a longer DUP) presented an higher risk to become resistant patients. In line with previous findings, Demjaha et al. (8) reported that longer DUP predicted treatment resistance. Moreover, Yoshimura et al. (76) found that a shorter DUP predicted favorable response and remission in FEP patients with schizophrenia.

All literature findings highlighted the importance to detect psychosis at onset and to early consider treatment-related factors because they are modifiable risk variables.

## Discussions

There is a general consensus among authors in retaining that the identification of specific factors predicting treatment response in patients with FEP significantly ameliorates the quality of clinical management of these patients in the critical early phase of pathology. Adequate early interventions produce a positive effect on long-term illness outcome, in terms of remission and recovery.

Main findings of our review show that among patient-related predictors of resistance to treatment lower premorbid functioning is an important factor in distinguishing antipsychotic non-responders from responders. Lower educational level can be also considered as a robust predictor of resistance, while the role of age and marital status is still controversial. Several studies suggest the male gender as a potential risk factor for treatment non-response, but we have to consider that many other studies have not confirmed the effect of gender.

Regarding disease-related predictors of treatment resistance, the higher level of negative symptoms from the FEP and their persistency over time induces a worse impairment of social functioning, more serious psychopathological phenomena, and a higher degree of refractoriness to treatment then controls who present a progressive decrease of negative symptoms. Positive, disorganized, and cognitive symptoms seem to be less significant in predicting treatment response. According to our review, the two main predictors of resistance related to the disorder are the diagnosis of schizophrenia and the younger age at onset. Among comorbidity conditions substance use disorder is the most studied predictive factor of treatment resistance and poor outcome.

Some neurobiological and neuroimaging factors may be identified as potentially involved in the mechanisms of response/non-response to therapies, but none of these factors has been identified as reliable predictor that can allow to separate responders and resistant patients in the course of the FEP. Some literature data support the hypothesis that the level of the dopamine synthesis is a potential biomarker of responsiveness to treatment. In addition, blunted cortisol awakening response and higher concentrations of pro-inflammatory cytokines are biological predictive factors of treatment resistance in early stages of psychotic disorders. Some neuroimaging studies show that at first stages of illness patients who do not respond have a significant reduction of pituitary volume and defects of cortical folding. Few innovative investigations have explored potential genetic predictors of treatment resistance, but initial data do not allow to draw any conclusion. Genetic studies about response to medications in early phases of psychosis are required, considering that only few initial investigations with inconclusive results are available.

Finally, lack of adherence to prescriptions, no early response (within 2 weeks) to antipsychotics, and prolonged of duration of untreated psychosis are the most important treatment-related factors that predict resistance.

Data provided from cited studies are displayed in Tables 1, 2.

**Table 1:** Summary of studies on predictive factors of treatment resistance.

	**Study design**	**Patients (*n*)/type**	**Trial duration**	**Predictors of**
**PATIENT-RELATED FACTORS**				
Malla et al. (44)	Longitudinal study	107 FEP SZ	2 years	**Response/remission** Better premorbid adjustment
Emsley et al. (45)	Longitudinal study	57 FEP SSD	2 years	**Non-response/resistance** Single status, lower premorbid functioning
Crespo-Facorro et al. (40)	RCT olanzapine vs. haloperidol vs. risperidone	172 FEP SSD	6 weeks acute phase (in the context of 3 years longitudinal intervention)	**Non-response/resistance** lower premorbid functioning
Selten et al. (54)	Longitudinal study	125 FEP SZ	30 months	**Non-response/resistance** Male gender (plus substance use)
Albert et al. (43)	Longitudinal study	255 FEP	5 years	**Response/remission** Female gender, higher age, good premorbid function
Addington and Addington (42)	Longitudinal study	240 FEP SSD	36 months	**Non-response/resistance** Reduced social functioning and lower premorbid functioning
Levine et al. (46)	Longitudinal study	263 SSD at recent onset	2 years	**Response/remission** Good premorbid functioning
Derks et al. (55)	Randomized, open-label, prospective study olanzapine vs. haloperidol vs. risperidone	498 FEP SSD	1 year	**Non-response/resistance** Male gender
Verma et al. (50)	Naturalistic study	1,175 FEP SSD	2 years	**Response/remission** Female gender, tertiary education
Teferra et al. (65)	Longitudinal study	312 FEP SZ	5 years	**Response/remission** Single status
Crespo-Facorro et al. (41)	RCT olanzapine vs. haloperidol vs. risperidone	375 FEP SSD	6 weeks	**Non-response/resistance** Poorer premorbid adjustment
Díaz et al. (51)	Randomized, open-label, prospective study olanzapine vs. haloperidol vs. risperidone	174 FEP SSD	1 year	**Non-response/resistance** Male gender, single status, and low education level
Zhang et al. (53)	Prospective cohort study	398 FEP SZ	1 year	**Response/remission** Higher age
Di Capite et al. (52)	Longitudinal study	63 FEP SSD antipsychotic discontinuation	1 year	**Non-response/resistance** Male gender Not related with educational level
Wimberley et al. (49)	Cohort study	8,624 SZ at first hospital contact	9 years	**Non-response/resistance** Younger age, living in less urban area, low education level
Lally et al. (56)	Longitudinal study	246 FEP SSD	5 years	**Non-response/resistance** Male gender
Lasalvia et al. (48)	Retrospective study	444 FEP SSD	9 months	**Response/remission** Good premorbid adjustment and insight regardless of treatment, higher educational level
Friis et al. (79)	Longitudinal study	301 FEP SSD	10 years	**Non-response/resistance** Lower premorbid functioning
**DISEASE-RELATED FACTORS**				
Lambert et al. (69)	Retrospective study	367 FEP SSD	18 months	**Non-response/resistance** High positive symptoms
Milev et al. (67)	Longitudinal study	99 FEP SSD	7 years	**Non-response/resistance** Cognitive and negative symptoms
Siegel et al. (74)	longitudinal study	208 FEP SZ	2–8 years (mean 3 years)	**Non-response/resistance** High positive, negative, and depressive symptoms
Selten et al. (54)	Longitudinal study	125 FEP SZ	30 months	**Non-response/resistance** Substance use
Addington and Addinton (42)	Longitudinal study	240 FEP SSD	36 months	**Non-response/resistance** High positive and negative symptoms
Boter et al. (82)	Longitudinal study	498 FEP SSD	1 year	**Response/remission** Absence of use disorder
Strauss et al. (72)	Longitudinal study	56 FEP SZ	20 years	**Non-response/resistance** Deficit syndrome
Levine and Rabinowitz (47)	Longitudinal study	49 FEP SSD	2 years	**Non-response/resistance** Cognitive impairment Diagnosis of schizophrenia Early age at onset
Üçok et al. (62)	Longitudinal study	93 FEP SZ	2 years	**Response/remission** High positive and low negative symptoms at onset
Galderisi et al. (73)	RCT olanzapine vs. amisulpride vs. ziprasidone vs. quetiapina	345 FEP SSD	1 year	**Non-response/resistance** Persistent negative symptoms
Verma et al. (50)	Naturalistic study	1,175 FEP SSD	2 years	**Non-response/resistance** Diagnosis of schizophrenia
Crespo-Facorro et al. (41)	RCT olanzapine vs. haloperidol vs. risperidone	375 FEP SSD	6 weeks	**Non-response/resistance** High positive and disorganized symptoms, diagnosis of schizophrenia, and early age at onset
Gaebel et al. (78)	RCT risperidone vs. haloperidol	166 FEP SZ	1 year	**Response/remission** Low positive and negative symptoms
Pelayo-Terán et al. (81)	RCT risperidone vs. haloperidol	161 FEP SSD	6 weeks	**Non-response/resistance** Cannabis use
Austin et al. (70)	longitudinal study	496 FEP SSD	10 years	**Non-response/resistance** Negative symptoms Substance use
Chiliza et al. (7)	Longitudinal study	126 FEP SSD	1 year	**Non-response/resistance** High negative symptoms
Ventura et al. (75)	Longitudinal study	146 SZ recent onset	1 year + 7 years of follow-up	**Non-response/resistance** Early negative symptoms
Friis et al. (79)	Longitudinal study	301 FEP SSD	10 years	**Non-response/resistance** Diagnosis of schizophrenia
Wimberley et al. (49)	Cohort study	8,624 SZ at first hospital contact	9 years	**Non-response/resistance** Diagnosis of schizophrenia Substance use
Demjaha et al. (8)	Longitudinal study	323 FEP SSD	10 years	**Non-response/resistance** Negative symptoms at onset diagnosis of schizophrenia and early age at onset
Yoshimura et al. (76)	Retrospective study	131 FEP SZ	Not reported	**Non-response/resistance** High negative symptoms
Downs et al. (77)	Cohort study	638 early-onset psychosis (10–17 years)	5 years	**Non-response/resistance** High negative symptoms
**NEUROBIOLOGICAL FACTORS**				
Garner et al. (89)	Controlled dose-finding study	42 FEP SZ with quetiapine	12 weeks	**Non-response/resistance** Larger pituitary volume
Palaniyappan et al. (90)	Case-control study	126 (80 FEP) SSD	12 weeks	**Non-response/resistance** Hypogyria at bilateral insular, left frontal, and right temporal regions
Mondelli et al. (88)	Longitudinal study	68 FEP 57 controls	12 weeks	**Non-response/resistance** Blunted cortisol awakening response and increased proinflammatory cytokines
Kim et al. (86)	RCT	12 SZ—TR with clozapine vs. 12 SZ responders vs. 12 healthy controls	12 weeks	**Non-response/resistance** Lower dopamine synthesis capacity in striatum
Walker et al. (87)	RCT (post-mortem)	14 SZ (6 TR) 9 healthy controls	Not available	**Non-response/resistance** Reduction of myelination in substantia nigra
**TREATMENT-RELATED FACTORS**				
Bottlender et al. (101)	Longitudinal study	58 FEP SZ	15 years	**Non-response/resistance** Longer DUP
Correll et al. (93)	Open-label study	131 acute SSD with fluphenazine	4 weeks	**Non-response/resistance** Poor response at week 1
Malla et al. (44)	Longitudinal study	107 FEP SZ	2 years	**Response/remission** Higher level of adherence
Leucht et al. (94, 95)	Data analysis from 7 RCTs	1,708 SSD	Not available	**Non-response/resistance** Poor response at week 1 and 2
Kinon et al. (26)	Data analysis from 5 RCTs	1,077 SSD	6 months	**Non-response/resistance** Poor early response (but early response does not predict subsequent response)
Boter et al. (82)	Longitudinal study	498 FEP SSD	1 year	**Response/remission** Higher level of adherence
Kinon et al. (96)	RCT	628 SSD with risperidone. If non response switch to olanzapine	12 weeks + 10 weeks if non early response	**Non-response/resistance** Poor response within 2 weeks
Üçok et al. (62)	Longitudinal study	93 FEP SZ	2 years	**Response/remission** Higher level of adherence
Zhang et al. (53)	Prospective cohort study	398 FEP SZ	1 year	**Response/remission** Higher level of adherence
Austin et al. (70)	Longitudinal study	496 FEP SSD	10 years	**Non-response/resistance** Longer DUP
Friis et al. (79)	Longitudinal study	301 FEP SSD	10 years	**Non-response/resistance** Longer DUP
Demjaha et al. (8)	Longitudinal study	323 FEP SSD	10 years	**Non-response/resistance** Longer DUP
Yoshimura et al. (76)	Retrospective study	131 FEP SZ	Not reported	**Response/remission** Shorter DUP

*SZ, schizophrenia; SSD, schizophrenia spectrum disorders; FEP, first episode of psychosis; TR, treatment resistant; RCT, randomized-controlled study; DUP, duration of untreated psychosis*.

**Table 2:** Clinical predictive factors of treatment resistance.

**Stable factors**	**Changeable**
Poor premorbid functioning	Lower educational level
Male gender	Single marital status
Younger age at onset	Negative symptoms
Diagnosis of schizophrenia	Substance use disorder
Neurobiological factors	Non-adherence
	Early non-response (within week 2)
	Duration of untreated psychosis

## Conclusions and Remarks From the Systematic Reviews

In literature we have examined five systematic reviews that focus on the topics of our investigation and can contribute to support our conclusions (20, 21, 32, 102, 103). In summary, these studies demonstrate that outcomes for patients with schizophrenia spectrum disorders can be significantly improved ameliorating early treatments and shortening the period of time that divides the beginning of symptoms from adequate specific interventions. Authors highlight that available trials are affected by some criticalities that are at least partly responsible for the heterogeneity of findings. For example, studies vary considerably in defining diagnosis of patients who can be enrolled (some authors include subjects with unspecified psychosis or brief psychotic disorder that can present a completely different course and outcome from schizophrenia). The diagnostic instability intrinsic to first episode has to be considered as potential bias for the results of investigations. In addition, few studies are designed with a sufficient statistical power to measure the predictive effect of several clinical factors with respect to treatment response. Studies have different duration and frequency of assessments during follow-up. Another very important limitation is that, as we have explained in the introduction of this review, criteria for determining resistance are different among studies, sometimes limited to severity of symptoms, in other cases extended to cognitive performances and social functioning. Despite these limitations and persisting uncertainty on the actual role of most clinical and biological factors, there is no doubt that developing research and refining knowledge on predictors of response in the early stage of psychotic disorders can produce noticeable results in terms of improvement of long-term clinical and functional outcome. In particular, some key predictive factors, like duration of untreated psychosis, or non-adherence to medications, can be modified by early intervention with significant effects on long-term outcome.

## Author Contributions

PB and SB equally contributed to revise studies in literature and to write the manuscript. PR contributed to project the review and to organize the structure of manuscript and tables.

### Conflict of Interest Statement

The authors declare that the research was conducted in the absence of any commercial or financial relationships that could be construed as a potential conflict of interest.
